# A Fast Inspection of Tool Electrode and Drilling Depth in EDM Drilling by Detection Line Algorithm

**DOI:** 10.3390/s8084866

**Published:** 2008-08-21

**Authors:** Kuo-Yi Huang

**Affiliations:** Department of Mechatronic Engineering, Huafan University, Taipei, Taiwan; E-mail: kyhuang@cc.hfu.edu.tw

**Keywords:** Electrical discharge machining (EDM), Electrode length, Drilling depth, Detection line algorithm (DLA), Peak decision method

## Abstract

The purpose of this study was to develop a novel measurement method using a machine vision system. Besides using image processing techniques, the proposed system employs a detection line algorithm that detects the tool electrode length and drilling depth of a workpiece accurately and effectively. Different boundaries of areas on the tool electrode are defined: a baseline between base and normal areas, a ND-line between normal and drilling areas (accumulating carbon area), and a DD-line between drilling area and dielectric fluid droplet on the electrode tip. Accordingly, image processing techniques are employed to extract a tool electrode image, and the centroid, eigenvector, and principle axis of the tool electrode are determined. The developed detection line algorithm (DLA) is then used to detect the baseline, ND-line, and DD-line along the direction of the principle axis. Finally, the tool electrode length and drilling depth of the workpiece are estimated via detected baseline, ND-line, and DD-line. Experimental results show good accuracy and efficiency in estimation of the tool electrode length and drilling depth under different conditions. Hence, this research may provide a reference for industrial application in EDM drilling measurement.

## 1. Introduction

Electrical discharge machining (EDM) is a nontraditional machining method usually employed in production of die cavities via the erosive effect of electrical discharges between a tool electrode and a workpiece. Its unique feature of using thermal energy to machine electrically conductive materials irrespective to their hardness and toughness make it applicable not only in the manufacture of die and mold components, but also in the fields of medicine, automotive and aerospace industry. The advantage with EDM is its ability to machine complex cavities, small parts with sharp internal or external radii, and fragile materials or workpieces requiring the creation and modification of very thin walls. However, the occurrence of tool electrode wear is unavoidable and is a very critical issue since tool shape degeneration directly affects the final shape of die cavity. To improve the machining accuracy in the geometry of a workpiece, methods to detect the tool electrode wear as well as compensate the wear of the tool electrode are required. Existing tool wear sensing methods such as offline wear prediction [1, 2] and real-time tool wear sensing approaches based on discharge pulse evaluation [3-5] are all indirect methods.

Optical measurement methods using machine vision techniques have been used for real-time tool wear evaluation in conventional machining processes [6-9]. Jurkovic *et al.* [6] used a machine system with a laser diode to measure the profile deepness of workpieces whereby a 3D image of a relief surface was obtained. Kim *et al.* [7] developed a system with a CCD camera and an exclusive jig to measure the size of tool wear in a direct manner. Sortino [8] established a tool condition monitoring system which can detect the worn area of tool. Methods such as statistical, high pass, saturation, and normalization filters were used to detect the surface profile and worn area. Kwon and Fischer [9] proposed a machine vision system that can measure the worn area of cutting tool in lathe machining process.

Kaneko *et al.* [10] suggested an optical measurement method to measure the tool electrode deformation in the EDM process. The dielectric fluid droplet on the tool electrode had to be blown off first by compressed air after the electrode was pulled up to a position where the machine vision system operated. A shadow image of electrode was then acquired using a parallel light and the deformation of the tool electrode was estimated. Nevertheless, such method can only measure the deformation of tool electrode. The system is incapable of measuring the drilling depth in workpieces because the surface color of the electrode is dark under the parallel light source.

Currently, the drilling depth of a workpiece is not measured directly online in the EDM drilling process but, in fact, manually measured after dissecting the workpiece. To date, no studies have attempted to measure the drilling depth and the tool electrode length (with the dielectric fluid droplet on it) at the same time in EDM. Thus, this paper will present a new approach - a detection line algorithm that detects the electrode length with dielectric fluid droplet on it and the drilling depth in workpieces rapidly and effectively.

The organization of this paper is as follows. Section 2 describes the basic analysis and the proposed method for detecting the tool electrode length and drilling depth. Experimental results and discussions are given in Section 3 that confirm the effectiveness of the proposed method. Section 4 will present some concluding remarks.

## 2. Materials and Methods

### 2.1 Image Acquisition System and EDM Drilling Machine

**Figure 1.**
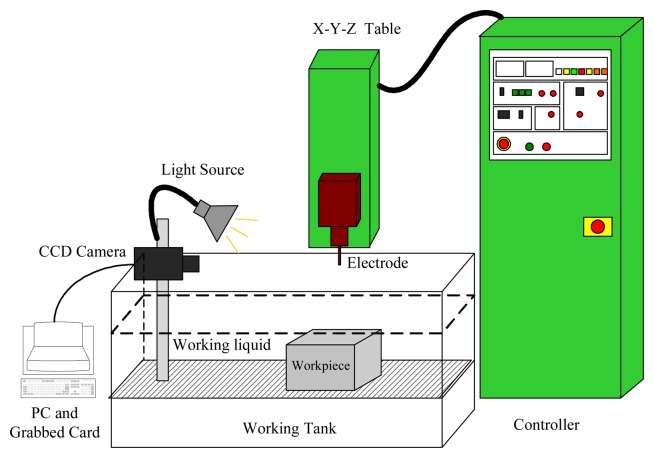
Experimental configuration

As shown in Figure 1, an EDM machine (JM-430, Jiten) and a controller (P-30, Jiten) were employed to drill workpieces to certain depths. In order to obtain tool electrode images on-line, a machine vision system is proposed. Images were acquired using a CCD (coupled-charge device) monochrome camera (WAT-902B, Watec) with a zoom lens (Zoom 6000 combinations, Navitar), a frame grabber (Meteor, Matrox Inc), and a personal computer (Intel Pentium 4 processor 2.4 GHz). Matrox Imaging Library (MIL 8.0, Matrox Inc) was linked to the programs to grab monochrome images of 640×480 pixels in size. The CCD camera was employed for image acquisition with forward lighting. Images were stored in the hard drive of the personal computer in tagged image file format (TIF). Image processing was performed using Visual C++ 6.0 programming.

### 2.2 Electrode Length and Drilling Depth Detection

#### 2.2.1 Electrode Image Extraction

**Figure 2.**
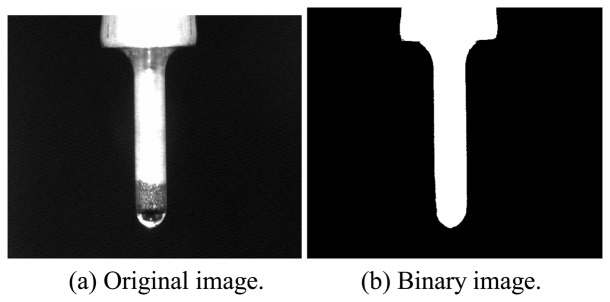
Electrode image.

**Figure 3.**
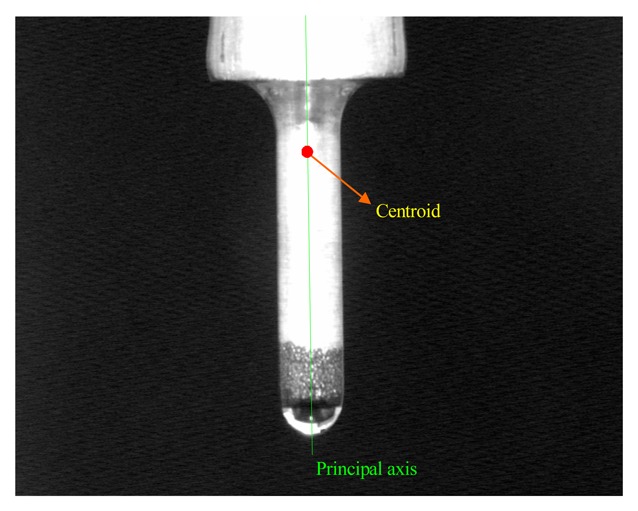
The principle axis and centroid of electrode.

Segmenting the tool electrode image is an essential procedure once the characteristics of the electrode have been extracted. The electrode image is extracted using thresholding, hole-filling, closing, and opening operators, as illustrated in Figure 2. Firstly, the principle axis of the tool electrode needs to be identified. By assuming the binary image of electrode is *f* (*x_i_*, *y_i_*) (where *i=1, 2,…N*, and the total number of pixels is *N*), the centroid is obtained as $\bar{X}=\frac{1}{N}\sum_{i}^{N}x_{i},\bar{Y}=\frac{1}{N}\sum_{i}^{N}y_{i}$. The covariance matrix is defined as $C=\frac{1}{N}\sum_{i}^{N}V_{i}V_{i}^{T}-MM^{T}$, in which *V_i_* is the *i*'th coordinate vector of the image and $M=\frac{1}{N}\sum_{i}^{N}V_{i}$ is the mean vector. *T* indicates vector transposition. A pair of orthogonal eigenvectors of the covariance matrix is calculated. The geometric features – the centroid and the principle axis of electrode, are then computed using eigenvectors, as shown in Figure 3.

#### 2.2.2 Detection Line Algorithm

**Figure 4.**
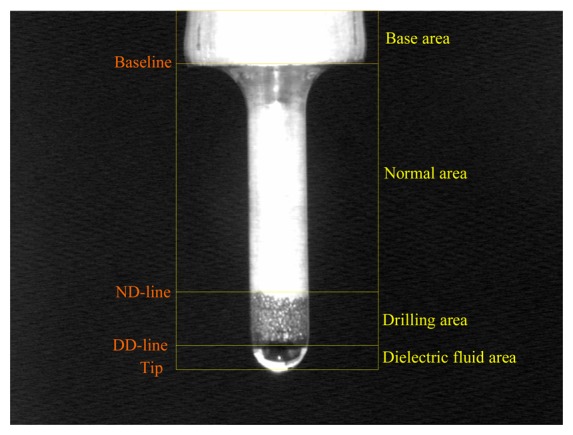
Baseline, ND-line, and DD-line

Before describing the detection line algorithm in detail, we first consider the basic analysis in a situation that the tool electrode image is well defined. As indicated in Figure 4, significant areas of the tool electrode include a base area, normal area, drilling area, and dielectric fluid droplet on the electrode tip. However, these areas are difficult to distinguish accurately and rapidly. Different area boundaries are defined: (1) a baseline between the base and normal areas, (2) a ND-line between the normal and drilling areas, and (3) a DD-line between the drilling area and dielectric fluid droplet.

**Figure 5.**
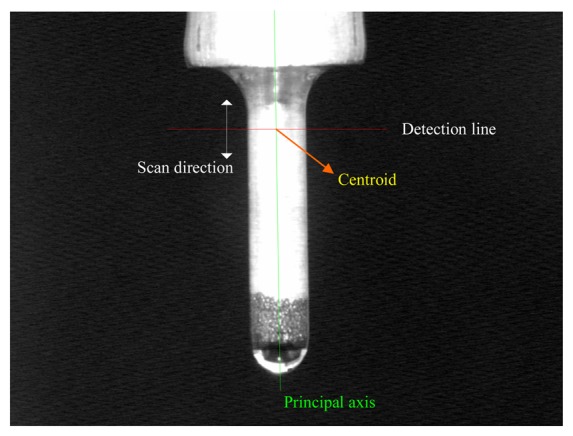
Scan direction of detection line on electrode.

**Figure 6.**
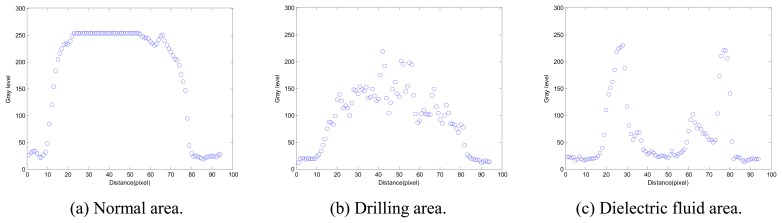
The distributions of gray levels on a detection line.

**Figure 7.**
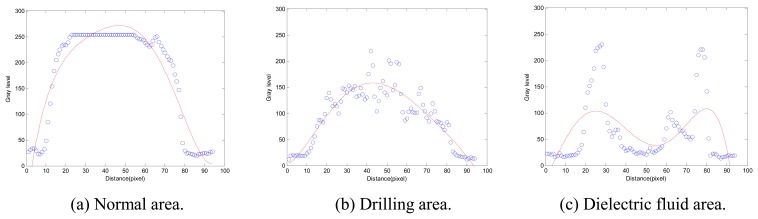
Fitted curve for gray level on a detection line.

A prior experiment proceeded as follows. A detection line (100 pixels in length) is used to find gray levels along the direction of the principle axis from the centroid to the tip of the electrode section with scanning resolution in a pixel, as shown in Figure 5. There are different distribution forms of gray levels in different areas, as illustrated in Figure 6. The distributions of gray level are around 255 on the glossy normal area, as shown in Figure 6(a). In Figure 6(b), the distributions of gray level are between 100 and 255 because there are irregular texture images on the drilling area. As the electrode tip (with dielectric fluid droplet on it) is illuminated and thus causing a reflection of the dielectric fluid droplet, a dual peak appears in Figure 6(c), and the distributions of gray levels are between 0 and 255 on the dielectric fluid droplet. Therefore, a polynomial curve fitting method is adopted to fit the distributions of gray levels on detection lines. In Figure 7, the form of fitted curve is a single peak and the maximum value (*N_max_*) is greater than 255 on the normal area. The fitted curve is also a single peak on the drilling area, but the maximum value (*T_max_*) is far smaller than *N_max_*. There is a special property – the dual peak on the dielectric fluid droplet area.

However, the distribution of gray levels method is not applicable in determining the position of the baseline because variations in gray levels along a detection line from normal to base areas are unobvious. For this reason, the baseline is determined using a detection line according to an obvious variation of the diameter from the normal area to the base area. Therefore, a novel method – the detection line algorithm (DLA) is proposed to find the positions of the ND-line, DD-line, and baseline. These boundary lines are then used to segment the base, normal, drilling areas, and dielectric fluid droplet on the tool electrode according to the above-mentioned contentions in this study. Thus, this method can be employed to estimate the tool electrode length with the dielectric fluid droplet remains on its tip effectively.

DLA is described as follows:

*Step 1*: Compute the distribution function of gray level defined as
  (1) $$g\left(p,t\right)=a_{0,p_{i}}+a_{1,p_{i}}t+a_{2,p_{i}}t^{2}+a_{3,p_{i}}t^{3}+a_{4,p_{i}}t^{4}+a_{5,p_{i}}t^{5}$$
  by a polynomial curve fitting method [11], where *p* = {*p_1_, p_2_, …, p_n_*} is a position parameter of the detection line along the principle axis of the tool electrode centroid and *t* is a distance variable with the detection line.
*Step 2*: Find the local maximum value *g_max_*(*p_i_,t*) according to *g*(*p_i_,t*). Further, the function of local maximize value *g_max_*(*p,t*) is established by obtaining *g_max_*(*p_i_,t*).
*Step 3*: Determine the boundaries:

(1) Baseline
  The diameter function of electrode *d*(*p*) is established by a detection line. The first-order derivatives of *d*(*p*) is defined as
  (2) $$d^{\prime }\left(p\right)=\frac{\Delta d}{\Delta p}=\frac{d\left(p_{i+1}\right)-d\left(p_{i}\right)}{p_{i+1}-p_{i}}$$
  Thus, the location of baseline is obtained at the point *p_max_* as maximum value of |*d*′(*p*)|. It means that an obvious variation of the diameter is at the baseline.
(2) ND-line
  The slope variation *m*(*p_i_*,*t*) of *g_max_* (*p_i_, t*) is defined as
  (3) $$m\left(p_{i},t\right)=\frac{\Delta g_{\max}}{\Delta p}=\frac{g_{\max}\left(p_{i+1},t\right)-f(g_{\max}\left(p_{i},t\right)}{p_{i+1}-p_{i}}$$
  Therefore, the position of ND-line can be found at point *p_ND_* as the first obvious variation |*m*(*p_ND_*,*t*)| *≥ δ* on scanning the tool electrode. *δ* is a positive constant.
(3) DD-line
  The peak decision method (PD method) is based on the peak number (*PN*) of *g*(*p_i_,t*) moving along the tool electrode in this study. The location of DD-line is detected by PD method when *PN* is alternated from 1 (a single peak) to 2 (a dual peak). *PN* is defined as
  (4) $$PN=\{\begin{array}{lll} 1, & \text{then g}\left(p_{i},t\right)\in & \text{normal or drilling areas}\\ 2, & \text{then g}\left(p_{i},t\right)\in & \text{the drop of dielectric fluid} \end{array}$$
*Step 4*: The tool electrode length and drilling depth are defined as:
  (1) Tool electrode length is the distance between the baseline and ND-line.
  (2) Drilling depth is the distance between the ND-line and DD-line.

The algorithms for the tool electrode length and detection and computation are developed using image processing techniques and DLA in this study.

## 3. Results and Discussion

**Figure 8.**
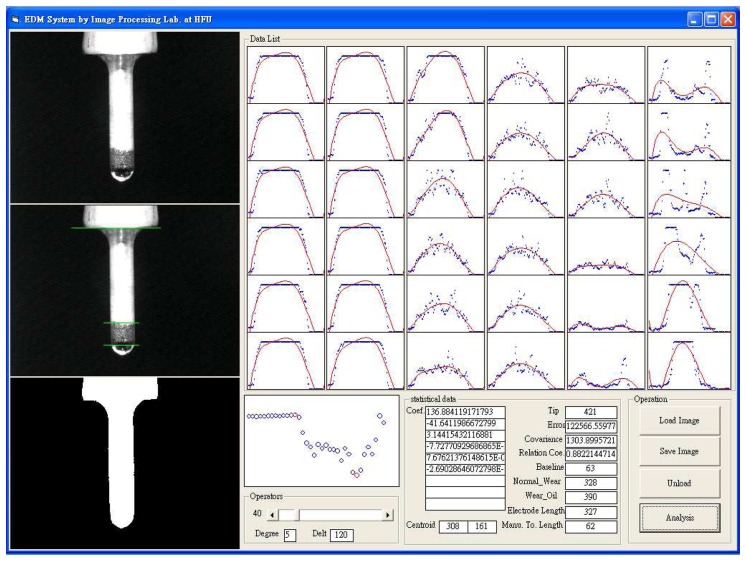
The EWM software interface.

In this study, the electrode wear detection (EWD) software is written using image processing techniques, DLA in Visual C++ 6.0, and MIL 8.0 library, as shown in Figure 8. The EWD software extracts a binary tool electrode image (threshold value is 80), and identifies the centroid and principal axis of the electrode. Further, the EWD software can estimate the tool electrode length and drilling depth in a workpiece accurately and rapidly.

**Figure 9.**
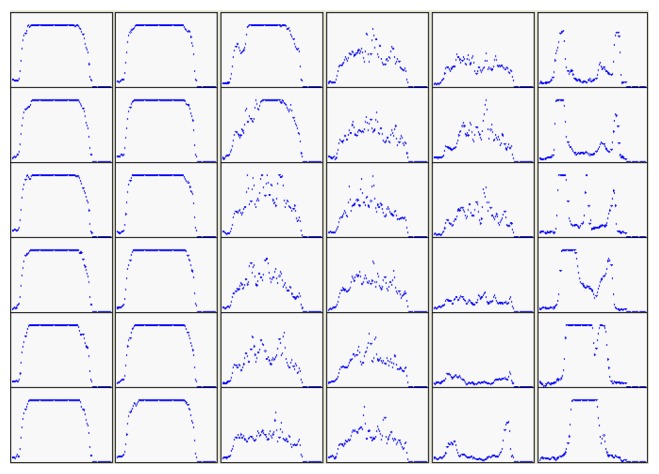
The gray level distributions on detection lines.

The distributions of gray levels from the centroid to the tip of the tool electrode using a detection line along the principle axis are illustrated in Figure 9.

**Figure 10.**
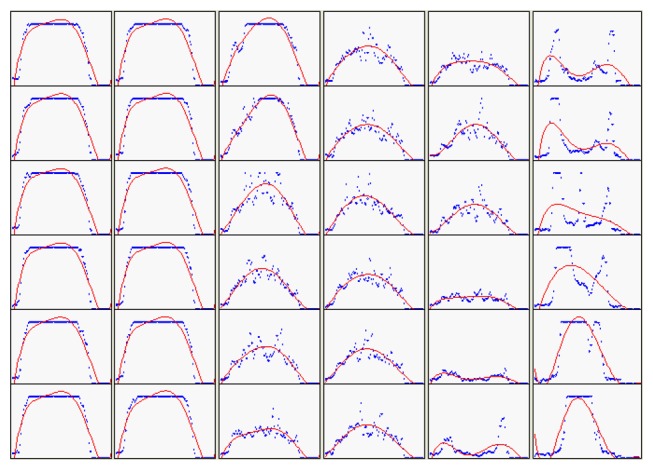
The gray level curves on detection lines.

**Figure 11.**
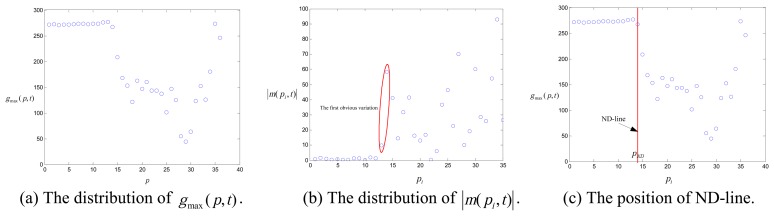
The ND-line detecting process. (with *p* as a position parameter)

In Figure 10, the gray level curves *g*(*p*,*t*) are fitted by polynomial curve fitting which automatically uses the 5th order of polynomial function. The local maximum g_max_ (p,t) have been found for *g*(*p*,*t*), as shown in Figure 11(a). The slope *m*(*p_i_*,*t*) of *g*_max_ (*p*,*t*) is obtained by Eq.(3). Thus, ND-line is determined in accordance with the first obvious variation of |*m*(*p_i_*,*t*)| as indicated in Figure 11(b) and 11(c).

**Figure 12.**
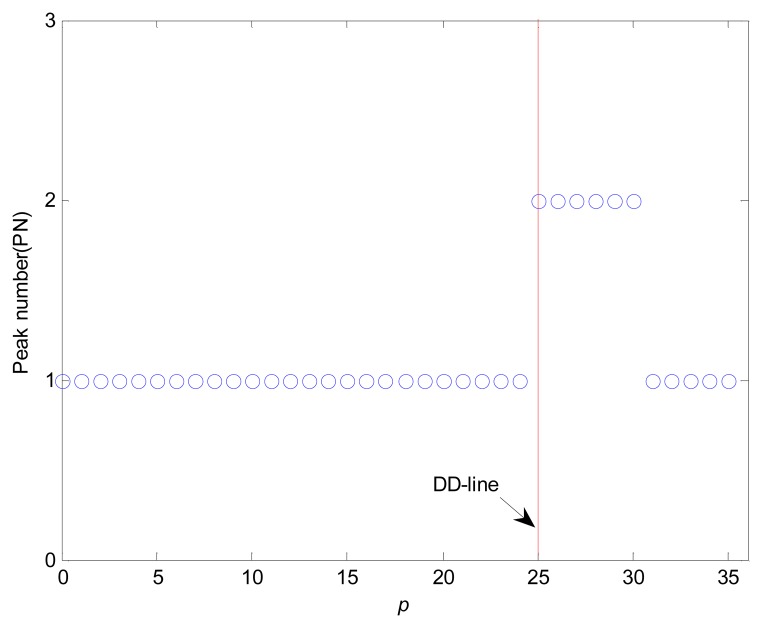
The distribution of peak number and DD-line using PD method (with *p* as a position parameter).

The location of DD-line is estimated using PD method when the peak number (*PN*) is alternated from 1 (a single peak) to 2 (a dual peak). The peak number of gray level curve *g*(*p*,*t*) is shown in Figure 12. Although the gray levels of the drilling area scatter in distance-gray level space, the distribution of gray levels appears as a single peak curve. For the dielectric fluid droplet, the fitted curve is a dual peak. The shapes and maximum values of fitted curves are contributed to detections of the ND-line and DD-line positions.

**Figure 13.**
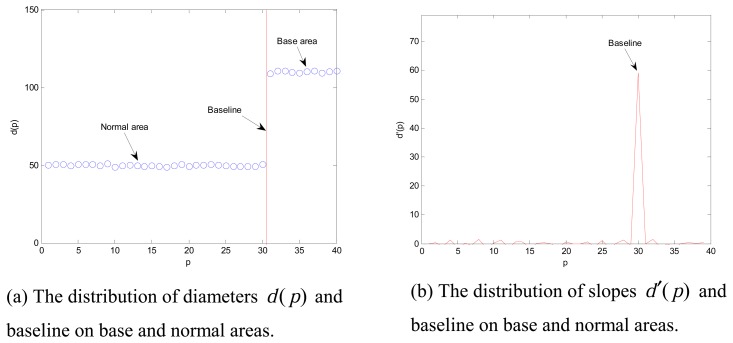
The baseline detecting process. (with *p* as a position parameter)

Finally, for the detection of the baseline, the diameter function of electrode *d*(*p*) and its first-order derivative *d*′(*p*) are obtained using DAL, as showed in Figure 13. Hence, the position of baseline is determined accurately.

**Figure 14.**
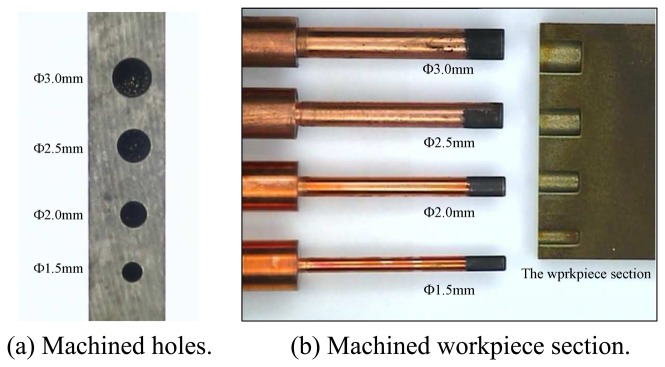
The drilled electrodes and a workpiece section.

Experimental conditions are shown in Table 1. Copper is used as the tool electrode anode, and the diameters of electrode are 1.5 mm (20 samples), 2.0 mm (20 samples), 2.5 mm (20 samples), and 3.0 mm (20 samples) respectively. A series of experiments with different machining parameters are performed and EDW is employed to estimate the tool electrode lengths and drilling depths. The workpieces are then cut open by WEDM (wire electrical discharge machine) manually for actual drilling depths measurement. The estimated values of electrode length and drilling depth are obtained using our machine vision system, and manually measured values are obtained by a tool-maker microscope. Figure 14 shows the machined holes, electrodes and cut section of the workpiece. Experimental results are given in Table 2. The drilling speed is increased with the peak current, but the electrode wear (i.e., the difference in electrode length before and after the drilling) is augmented simultaneously. For example, the average machined time is 28 minutes (electrode wear 0.062 mm) in a peak current of 6.0 A, but slowed down to 42 minutes (electrode wear 0.033 mm) in a peak current of 5.0 A with the same drilling depth (2.0 mm). The average error between estimated and manually measured electrode lengths is 0.075 mm, and for drilling depths is 0.245 mm. The accuracy of estimated drilling depth is reduced because the accumulating carbon on the electrode surface grown at the drilling area, and the size and shape of dielectric fluid droplet is uncertain. For this reason, the boundary between the normal and drilling areas is an irregular border. Moreover, another possible cause is the gap distance in EDM drilling. In general, the gap distance is an uncertain variable. However, under certain circumstances, the gap distance is more precise, and hence, overall accuracy can be increased. For example, a steady gap status is achieved when using the servo feed control in EDM drilling. Thus, the gap distance (about 0.05 mm) can be taken into account to estimate the drilling depth. The error of measured drilling depth is then reduced to 0.195 mm.

According to the above-mentioned results and discussion, the advantage of the proposed method is summarized as follows: the tool electrode length and the drilling depth of workpieces can be estimated effectively (no need of an air compressor to blow off the dielectric fluid droplet and cut open the workpieces).

However, the weak point of this study is that round corner of tool cannot be measured because of the retaining dielectric fluid droplet on the tip of tool electrode. Our expectancy is achieved according to the experimental results. The results of this study may be of interest to EDM industry attempting to develop an inspector and to increase efficiency.

## 4. Conclusions

The objective of this study is to propose a novel and efficient method for sensing electrode parameters in EDM drilling process. As presented in this paper, the conclusions can be summarized as follows: (1) the proposed detection line algorithm (DLA) can detect the tool electrode length and drilling depth accurately and effectively. DLA includes a polynomial curve fitting, slope variation, and PD methods, (2) our machine vision system is powerful and flexible under different experimental conditions on real time measurement, (3) our system can provide an important reference for EDM industry.

## Figures and Tables

**Table 1.** Experimental conditions.

Parameters	Values
Electrode material	Copper
Peak current	5.0A, 6.0A
Open voltage	90 V
Pulse duration	100 μs
Pulse interval	100 μs
Diameter of tool electrode	Ø 2.0, 2.5, 3.0 mm
Workpiece material	SKD11
Gap distance	0.05 mm
Dielectric flushing method	Static condition
Machined depth	1.5, 2.0, 2.5, 3.0 mm

**Table 2.** Estimated and manual results after EDM drilling.

No.	Machining condition		Electrode diameter	Ave. machining time (min.)	Ave. electrode length (mm)			Ave. Machined depth (mm)		

					Manual	Estimated	Error	Manual	Estimated	Error
1	Setting depth	2.0 mm	1.5 mm	42	19.85	19.76	0.09	2.08	1.95	0.13
			2.0 mm	61	19.96	19.89	0.07	2.08	1.97	0.11
	Peak current	5.0 A	2.5 mm	117	19.93	19.94	0.01	2.12	1.96	0.16
			3.0 mm	149	19.87	19.89	0.02	2.11	1.97	0.14

2	Setting depth	3.0 mm	1.5 mm	53	19.77	19.88	0.11	3.13	2.89	0.24
			2.0 mm	99	19.91	19.94	0.03	3.17	2.87	v0.30
	Peak current	5.0 A	2.5 mm	152	19.85	19.94	0.09	3.04	2.82	0.22
			3.0 mm	199	19.91	20.04	0.13	3.14	2.87	0.27

3	Setting depth	4.0 mm	1.5 mm	60	19.78	19.92	0.14	4.19	3.85	0.34
			2.0 mm	114	19.91	19.94	0.03	4.10	3.89	0.21
	Peak current	5.0 A	2.5 mm	163	19.78	19.84	0.06	4.21	3.81	0.40
			3.0 mm	217	19.92	19.94	0.02	4.29	3.89	0.40

4	Setting depth	5.0 mm	1.5 mm	74	19.97	19.84	0.13	5.05	4.92	0.13
			2.0 mm	133	19.88	19.90	0.02	5.10	4.96	0.14
	Peak current	5.0 A	2.5 mm	174	20.04	20.05	0.01	5.06	4.92	0.14
			3.0 mm	242	20.03	19.94	0.09	5.08	4.97	0.11

5	Setting depth	2.0 mm	1.5 mm	28	19.92	19.94	0.02	2.14	1.88	0.26
			2.0 mm	41	19.83	19.99	0.16	2.12	1.90	0.22
	Peak current	6.0 A	2.5 mm	72	19.95	19.89	0.06	2.13	1.86	0.27
			3.0 mm	104	19.99	19.84	0.15	2.11	1.89	0.22

6	Setting depth	3.0 mm	1.5 mm	36	19.88	19.94	0.06	3.14	2.87	0.27
			2.0 mm	65	19.86	19.84	0.02	3.19	2.88	0.31
	Peak current	6.0 A	2.5 mm	94	19.85	19.79	0.06	3.15	2.86	0.29
			3.0 mm	134	19.84	19.89	0.05	3.22	2.90	0.32

7	Setting depth	4.0 mm	1.5 mm	54	19.87	19.89	0.02	4.17	3.84	0.33
			2.0 mm	79	19.78	19.89	0.11	4.16	3.88	0.28
	Peak current	6.0 A	2.5 mm	115	19.87	19.79	0.08	4.18	3.91	0.27
			3.0 mm	187	19.93	19.79	0.14	4.20	3.90	0.30

8	Setting depth	5.0 mm	1.5 mm	65	19.82	19.94	0.12	5.16	4.89	0.27
			2.0 mm	107	19.87	19.84	0.03	5.19	4.92	0.27
	Peak current	6.0 A	2.5 mm	148	19.59	19.74	0.15	5.13	4.87	0.26
			3.0 mm	213	19.73	19.84	0.11	5.15	4.90	0.25

					Ave. error		0.075	Ave. error		0.245
